# A Systematic Review to Summarise and Appraise the Reporting of Surgical Innovation: a Case Study in Robotic Roux-en-Y Gastric Bypass

**DOI:** 10.1007/s11695-024-07329-8

**Published:** 2024-06-19

**Authors:** Marc M. Huttman, Alexander N. Smith, Harry F. Robertson, Rory Purves, Sarah E. Biggs, Ffion Dewi, Lauren K. Dixon, Emily N. Kirkham, Conor S. Jones, Jozel Ramirez, Darren L. Scroggie, Samir Pathak, Natalie S. Blencowe, on behalf of the RoboSurg collaborative group, Barry Main, Jane Blazeby, Sarah Dawson, Aimee Wilkinson, Annabel Jones, Aya Abbas, Benedict Turner, Charlie Thomas, David Henshall, Eleanor Boden, Emma Gull, Emma Sewart, Fergus Wood, Francesca Loro, Freya Hollowood, George Fowler, George Higginbotham, Grace Sellers, Ioan Hughes, Ishita Handa, Lorna Leandro, Louisa Paynter, Lucy Huppler, Lysander Gourbault, Manuk Wijeyaratne, Maximilian Dewhurst, Max Shah, Miraen Kiandee, Mo Dada, Oliver Brewster, Pat Lok, Rahul Winayak, Reesha Ranat, Ruby Lawrence, Ryan Millar, Sam Lawday, Sanjush Dalmia, Sophie Rozwadowski, Tanya Robinson, Teresa Perra, Tjun Wei Leow, Tom Brankin-Frisby, William Baker, William Hurst, Ysabelle Embury-Young, Abigail Vallance, Amber Young, Ben Zucker, Christin Hoffmann, Hollie Richards, James Olivier, Jonathan Rees, Keng Siang Lee, Rhiannon Macefield, Sian Cousins

**Affiliations:** 1 Bristol Centre for Surgical Research, Population Health Sciences, Bristol Medical School, University of Bristol, Canynge Hall, 39 Whatley Road BS8 2PS, Bristol, BS81QU UK; 2 University College Hospital, University College London Hospitals NHS Foundation Trust, London, NW12PB UK; 3 Peterborough City Hospital, Northwest Anglia NHS Foundation Trust, Peterborough, PE39GZ UK; 4 St Mary’s Hospital, Imperial College Healthcare NHS Trust, London, W21NY UK; 5 Southport and Ormskirk Hospitals NHS Trust, Southport, PR86PN UK; 6 Bristol Royal Infirmary, University Hospitals Bristol and Weston NHS Foundation Trust, Bristol, BS28HW UK; 7 St James’s University Hospital, Leeds Teaching Hospitals NHS Trust, Leeds, LS97TF UK

**Keywords:** Robotic surgery, Bariatric surgery, Roux-en-Y gastric bypass, Outcome reporting, Innovation, IDEAL framework

## Abstract

Robotic Roux-en-Y gastric bypass (RRYGB) is an innovative alternative to traditional laparoscopic approaches. Literature has been published investigating its safety/efficacy; however, the quality of reporting is uncertain. This systematic review used the Idea, Development, Exploration, Assessment and Long-term follow-up (IDEAL) framework to assess the reporting quality of available literature. A narrative summary was formulated, assessing how comprehensively governance/ethics, patient selection, demographics, surgeon expertise/training, technique description and outcomes were reported. Forty-seven studies published between 2005 and 2024 were included. There was incomplete/inconsistent reporting of governance/ethics, patient selection, surgeon expertise/training and technique description, with heterogenous outcome reporting. RRYGB reporting was poor and did not align with IDEAL guidance. Robust prospective studies reporting findings using IDEAL/other guidance are required to facilitate safe widespread adoption of RRYGB and other surgical innovations.

**Supplementary Information:**

The online version contains supplementary material available at 10.1007/s11695-024-07329-8.

## Introduction

Roux-en-Y gastric bypass (RYGB) is one of the most commonly performed bariatric procedures worldwide [1]. Laparoscopic RYGB (LRYGB) confers fewer post-operative complications and a shorter hospital stay than open surgery and is considered the ‘gold standard’ approach [2–5]. Despite its advantages, LRYGB is one of the most technically challenging laparoscopic surgical procedures to perform, predominantly due to difficulties in optimising port placement for both the gastro-jejunostomy and jejuno-jejunostomy, and the need for advanced surgical skills such as intracorporeal suturing and stapling [6, 7]. LRYGB can be associated with a prolonged learning curve of up to 100 cases before operating time and technical complications plateau [8].

Robotic technology is perceived to overcome the limitations of LRYGB, with advantages such as three-dimensional visualisation, improved surgeon ergonomics, superior tactile feedback, and easier manipulation of surgical instruments [9, 10]. A robotic platform was first used for RYGB in 2001, where the gastro-jejunostomy was undertaken robotically and the remainder through laparoscopic methods [11]. Since then, the technique has evolved with some surgeons adopting a totally robotic approach (RRYGB). Although this novel technique is becoming more widely adopted, uncertainties remain about whether it confers clinically meaningful benefits compared to laparoscopic approaches [12–15].

There is currently no requirement for innovative procedures such as RRYGB to undergo robust evaluation before widespread implementation. Historically, surgical innovation has been unstructured, unstandardised and lacking robust evidence from randomised controlled trials (RCTs) [16, 17]. The Cumberlege report (by the Independent Medicines and Medical Devices Safety Review) and reports from the Royal College of Surgeons of England (‘From innovation to adoption’ and ‘From theory to theatre: Overcoming barriers to innovation in surgery’) recommend that innovative surgical devices and procedures undergo rigorous testing prior to widespread adoption [18–20]. The Idea, Development, Exploration, Assessment, and Long-term follow up (IDEAL) framework, proposed in 2009, goes some way to achieve this. IDEAL describes a five-stage evaluation process to facilitate the robust evaluation of innovative surgical procedures and their safe introduction into routine clinical practice [21]. Evaluation occurs from first-in-human studies (stage 1), progressing to long-term studies for large-scale surveillance of outcomes (stage 4), with registry-based surveillance occurring at all IDEAL stages. The IDEAL framework also gives specific recommendations regarding the reporting of patient selection, study methodology, ethics, governance, surgeon expertise and outcome reporting. For example, patients are carefully selected according to narrowly defined criteria during stage 1. A wider range of patients become eligible as experience with the innovation accumulates during stages 2a, 2b and 3. Patient eligibility criteria are well-defined and more broadly inclusive by stage 4. Similarly, technical and safety outcomes are reported in stages 1–2 whereas by stage 4, patient-reported and health economic outcomes are required. A safe structure to objectively evaluate surgical interventions, such as the IDEAL framework, is therefore essential to ensure that comprehensive and robust evidence is generated. This then facilitates incremental learning, whereby researchers build on existing literature to create a reliable evidence base on which to safely introduce novel surgical procedures into clinical practice.

Whilst efforts have been made to evaluate the potential benefits of RRYGB, the quality of reporting and robustness of evaluation since its inception are yet to be investigated. This raises questions as to whether the reporting quality within currently available literature is sufficient to support widespread use of RRYGB. The aim of this study was to summarise and appraise the reporting of RRYGB as a case study of a surgical innovation in relation to the IDEAL framework.

## Methods

Methods were based on a previously published protocol for summarising and appraising the introduction of innovative surgical procedures and are summarised below [22]. Reporting was undertaken in accordance with PRISMA guidance (Supplementary Tables 1 and 2) [23].

### Search Strategy

Systematic searches were undertaken in Embase, Ovid Medline, the Cochrane Library and Web of Science, from inception to February 2024. Search terms for RYGB and robotic surgery were combined using the Boolean ‘AND’ operator (Supplementary Table 3).

### Selection Criteria

All primary research study designs (e.g. case reports, case series and comparative studies), reporting any outcomes for RRYGB as a treatment for obesity in adults aged 18 years or over, were eligible for inclusion. Studies were only included if RRYGB was the index bariatric procedure and was performed completely robotically. Studies reporting on initial robotic techniques where only some/one components of the procedure (e.g. gastrojejunostomy) was done robotically, were excluded. For comparative studies, only those comparing RRYGB with LRYGB, open RYGB or hybrid approaches were included. Conference abstracts, non-human and non-English language studies were excluded. Database studies were included as long as RRYGB was one of the index procedures and the outcomes for RRYGB were reported separately in the results section.

### Study Selection

After de-duplication, titles and abstracts were screened manually for eligibility by five independent reviewers (HR, NB and EK). Instances of non-consensus between reviewers were resolved via discussion. The same process was repeated for full text review.

### Data Extraction

Data from each paper was extracted by a member of the RoboSurg Collaborative and then verified by a senior team member using REDCap, a standardised bespoke online database [24, 25]. The following categories of data were extracted in accordance with IDEAL recommendations:

#### General Study Characteristics and IDEAL Stage

The study design, publication year, country of origin, number of included patients, number/type of included centres and IDEAL stage as reported by the authors were recorded. Where IDEAL stage was not provided, this was determined by two members of the review team (MH and HR) using an algorithm created by the IDEAL collaboration [26].

#### Governance and Ethical Factors

Information about ethical approval, funding statements, and conflicts of interest was extracted. Documentation of whether patients were specifically informed about the innovative nature of RRYGB was noted. The number of patients declining RRYGB, and their outcomes, were recorded where available.

#### Patient Selection and Demographics of Included Patients

Inclusion and exclusion criteria were extracted verbatim. Any reported modifications to patient selection criteria during the study, and reasons for these, were documented. The demographics of patients included in each study were extracted.

#### Surgeon Expertise and Training

The number and grade of surgeons participating in each included study was recorded, together with their previous experience with RRYGB. Details were extracted about any pre-specified criteria surgeons were required to meet, including any training. Strategies used by studies for monitoring surgical standards were recorded (e.g. proctoring, dual consultant operating). Information regarding surgeons’ learning curves was extracted verbatim.

#### Technique Description

Any methods used to describe the technique of RRYGB (e.g. written, photos, videos, references to other manuscripts) were recorded. To assess technique evolution, we recorded whether studies cited previously documented surgical techniques and/or provided updated descriptions, and any rationale for modifications to the previously described techniques.

#### Outcome Reporting

Individual outcomes from each study were extracted verbatim and coded by two independent reviewers (MH, HR) into one of eight pre-determined domains: technical, complications, laboratory and imaging, function and symptoms, trends and learning curves, health economic and resource utilisation, patient-reported and surgeon-reported (Table 1). The total number of unique outcomes across all studies was recorded. Outcomes were defined as unique if they were distinct compared to any other outcome. For example, outcomes with the same meaning but worded differently such as ‘length of stay’ and ‘duration of hospitalisation’ were not counted as unique. Where provided, the follow-up period for each recorded outcome was documented. Use of a core outcome set (i.e. an agreed minimum set of outcomes that should be reported in all clinical trials of a specific disease) was recorded. Reported outcomes were assessed for compliance with IDEAL guidance according to study stage.

**Table 1:** Outcome domains

Outcome domain	
Technical	Outcomes related to the operation or robot itself. This includes outcomes such as operative time, blood loss and conversions
Complications	Any deviation from the normal operative course. This included intra-operative complications and post-operative complications including readmission and reoperation, as well as mortality during the study period
Laboratory and imaging	Outcomes related to investigative results. This includes postoperative blood or imaging results
Function and symptoms	Outcomes related to post-operative weight loss, improvement in comorbidity status or symptoms
Trends and learning curve	Outcomes relating to the surgical learning curve including surrogate markers e.g. operative time per case number
Health and economic and resource utilisation	Outcomes relating to the cost or resource use of any aspect of the admission. This includes outcomes such as operative costs, wages and length of stay
Patient reported	Instruments such as satisfaction questionnaires and validated quality of life questionnaires, that must be completed independently by the patient
Surgeon reported	Outcomes related to the surgeons’ subjective experience of the robotic technology

### Data Synthesis and Analysis

Results were summarised using descriptive statistics and arranged chronologically where appropriate. An assessment was made of whether incremental and sequential progression through IDEAL stages had occurred. Finally, results were used to form a narrative summary. As the focus of this study was to summarise and appraise the reporting quality of RRYGB literature rather than to synthesise clinical outcomes, meta-analyses were not performed.

## Results

**Fig. 1:**
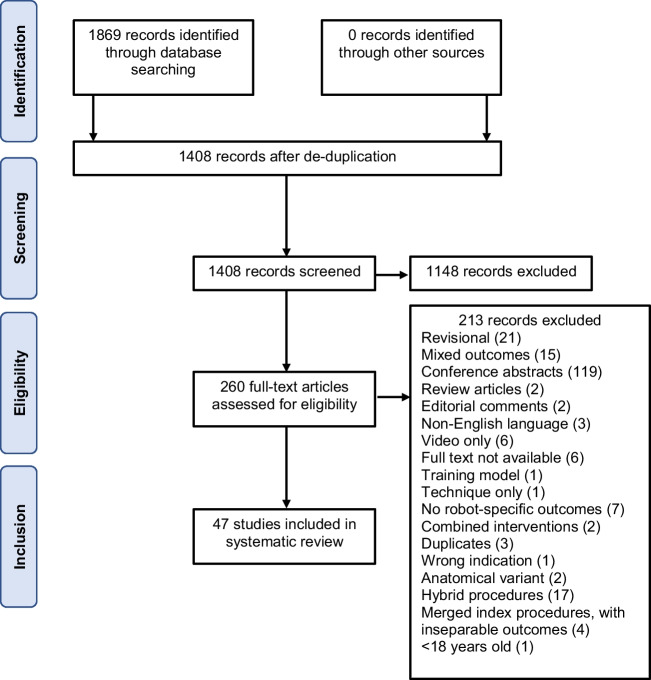
PRISMA diagram describing search strategy and details of excluded studies

After removing duplicates, searches yielded 1408 unique records, of which 260 full texts were screened for eligibility (Fig. 1). A total of 47 studies were included in the final analysis (Supplementary Table 4) [7, 9, 10, 12, 15, 27–68].

### General Study Characteristics and IDEAL Stage

Across the included studies, a total of 494,111 participants were included (median = 214, range = 1 -157,716) (Table 2). Although one study included 157,716 participants, 10 had fewer than 100 participants [67]. Studies were published between 2005 and 2024 and included one case report, 11 case series, 28 non-randomised comparative studies and seven retrospective analyses of registry database studies. Forty-one studies reported temporality of data collection, of which 34 were retrospective. Most studies (*n* = 27) were undertaken in the USA with none from the UK.

**Table 2:** Summary of study characteristics

Study characteristic	Number of studies reporting study characteristic *N* = 47 *n*(%)
Country of study	
United States	26 (55)
Switzerland	4 (9)
Netherlands	2 (4)
France	3 (6)
Greece	1 (2)
Italy	2 (4)
India	1 (2)
Germany	3 (6)
Spain	1 (2)
Turkey	1 (2)
Brazil	2 (4)
USA/Switzerland	1 (2)
Number of included patients	
1–10	3 (6)
11–50	3 (6)
51–100	6 (13)
> 100	35 (74)
Study design	
Case report	1 (2)
Case series	11 (23)
Non-randomised comparative study	28 (60)
Randomised controlled trial	0 (0)
Retrospective database analysis	7(15)
Study temporality	
Prospective	4 (9)
Retrospective	34 (72)
Mixed	3 (6)
Not reported	6 (13)
IDEAL stage*	
1	1 (2)
2a	6 (13)
2b	40 (85)
* IDEAL stages were only able to be assigned once temporality was ignored	
Number of centres	
1	34 (72)
> 1	7 (15)
Not reported	6 (13)
Centre type	
Specialist/tertiary/university	23 (49)
Mixed	3 (6)
General	2 (4)
Not reported	19 (40)

**Fig. 2:**
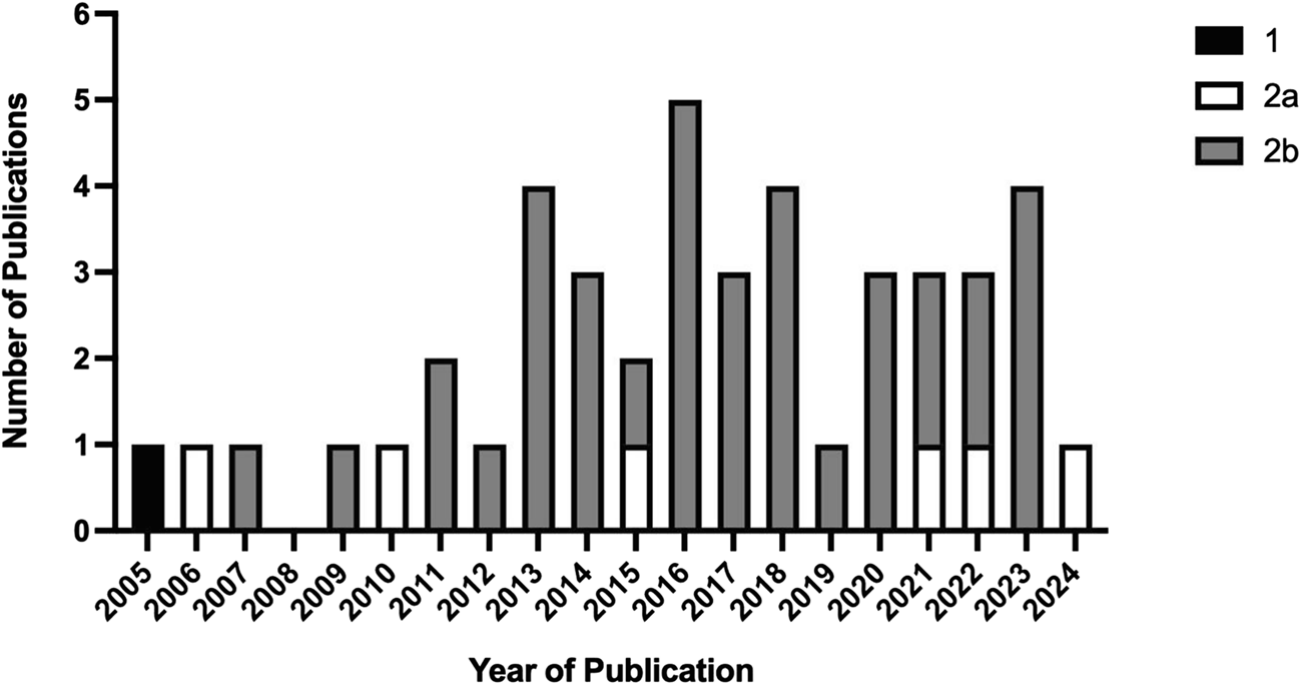
Bar chart showing publication dates and IDEAL stages of included papers. IDEAL stages were only able to be assigned once temporality was ignored

No studies reported an IDEAL stage. An IDEAL stage could only be accurately assigned to four prospective studies, because the IDEAL Collaboration’s algorithm requires them to be prospective in nature [26]. When ignoring the temporality of data collection, IDEAL stages were assigned as follows: stage 1 (*n* = 1, the first case report), stage 2a (*n* = 6) and stage 2b (*n* = 40). There was a lack of sequential progression through IDEAL stages as time progressed since 2005. This is evident from Fig. 2 due to the abundance of 2b studies, absence of stage 3/4 studies, lack of 2a studies performed early since the inception of RRYGB in 2005 and resurgence of 2a studies as time progressed.

### Governance and Ethical Factors

Eighteen studies reported obtaining ethical approval and nine reported exemptions. Reasons for this were not provided for most studies, however three studies stated exemption because the existing de-identified datasets used did not constitute human subject research. Only two studies stated that patients were specifically informed of the innovative nature of the intervention. Only one study provided information about the number of patients declining RRYGB.

A conflict-of-interest statement was provided for 33 studies, of which nine declared a conflict. Conflicts included i) involvement with a robotics company where one or more authors were a company representative (e.g. proctor, consultant, speaker or employee; *n* = 6), ii) non-financial support (e.g. teaching, research, training or equipment; *n* = 5) and iii) financial support (e.g. honoraria or personal fees; *n* = 5). Of the 10 studies not providing a conflict-of-interest statement, three reported receiving training from the robotics company.

### Patient Selection and Demographical Information

Thirty-five studies included information about patient selection criteria: 29 reported inclusion and 26 exclusion criteria (Table 3). No studies reported any changes to patient selection criteria during the study. A total of 44 patient characteristics were identified across the included studies, 30 of which were co-morbidities (Table 4). Most studies provided basic patient characteristics such as age (*n* = 45), sex (*n* = 40) and body mass index (*n* = 45), although no single characteristic was reported across all studies.

**Table 3:** Summary of patient selection criteria reported across the included studies

		Number of studies reporting patient selection criteria *N* = 47 *n*(%)
Number of studies reporting any selection criteria		35 (74)
Inclusion criteria		
Patient-related factors	Studies providing any inclusion criteria	29 (62)
	Age 18–80	5 (11)
	Age ≥ 18	3 (6)
	BMI > 40	6 (13)
	BMI > 35 with one comorbidity (HTN, T2DM and/or OSA)	6 (13)
	BMI 30–70	1 (2)
Operative factors	General Statement about meeting international criteria	9 (19)
	No previous abdominal procedures	1 (2)
	Primary RRYGB	6 (13)
Follow-up related factors	Minimum 30 days follow-up	2 (4)
	Minimum 12 months follow-up	1 (2)
	MBSAQIP registry patient	4 (9)
Exclusion Criteria		
Patient-related Factors	Studies providing any exclusion criteria	26 (55)
	Diagnosis of gastrointestinal dysmotility	1 (2)
	Pre-operative dialysis	1 (2)
	Absence of informed consent	1 (2)
	Anaesthetic Contraindications	3 (6)
	Hostile abdomen	2 (4)
	Pre-operative chronic opioid use	2 (4)
	No multimodal pain relief or TAP block given	1 (2)
	Double docking	1 (2)
	Other approaches (laparoscopic, hybrid, open)	7 (15)
Operative Factors	Concurrent procedures	8 (17)
	Non-stapling procedures	1 (2)
	Junior surgeon operating	1 (2)
	Previous bariatric procedure	15 (32)
	Conversion to different approach	3 (6)
	Number of cases performed by surgical team	2 (4)
	Previous antireflux surgery	1 (2)
	Missing registry data	4 (9)
	Coding error in registry data	1(2)
	Cases performed by gastroenterologist or interventional radiologist	1(2)

*BMI* body mass index; *HTN* hypertension; *T2DM* Type 2 Diabetes Mellitus; *OSA* obstructive sleep apnoea; *TAP block* Transabdominal Plane Block; *Double docking* the robot was undocked for a part of the procedure

**Table 4:** Summary of reporting of patient-related characteristics

		Number of studies reporting patient characteristic *N* = 47 *n*(%)
Demographics		
	Age	45 (96)
	Sex	40 (85)
	Ethnicity	6 (13)
Surgical related characteristics		
Weight-related	BMI	45 (96)
	Edmonton obesity staging	3 (6)
	Age of obesity onset	1 (2)
	Pre-operative weight	14 (30)
	Pre-operative height	4 (9)
Other	Previous abdominal surgery	15 (32)
	Liver volume	1 (2)
	Taking Steroids	1 (2)
	Taking Anticoagulants	4 (9)
	Leukocyte count	1 (2)
	Haemoglobin count	1 (2)
Comorbidities		
General co-morbidities	Comorbidities (not further specified)	6 (13)
	ASA grade	22 (47)
Cardiovascular	Angina	1 (2)
	Peripheral vascular disease	1 (2)
	Hypertension	21 (45)
	Hyperlipidaemia	20 (43)
	Previous MI or PCI	6 (13)
	Previous cardiac surgery	3 (6)
	Arrhythmia	1 (2)
Respiratory	Asthma	3 (6)
	OSA	21 (45)
	PE/VTE history	6 (13)
	Functional status	5 (11)
	COPD/other chronic lung disease	6 (13)
Endocrine	Diabetes	20 (43)
	IDDM	2 (4)
	NIDDM	3 (6)
	Metabolic syndrome	2 (4)
Other	Smoking	10 (21)
	Liver disease	2 (4)
	GORD	11 (23)
	CKD	4 (9)
	Osteoarthritis	6 (13)
	Depression	2 (4)
	Use of mobility device	2 (
	Dialysis	4
	Venous Stasis	1
	IVC filter	1
	Oxygen dependent	2
	Previous organ transplant	1

*VTE* venous thrombo-embolism; *IDDM* insulin-dependent diabetes mellitus; *NIDDM* non-insulin dependent diabetes mellitus; *PVD* peripheral vascular disease; *OSA* obstructive sleep apnoea; *GORD* gastro-oesophageal reflux disease; *CKD* chronic kidney disease; *ASA* American Society of Anaesthesiologists; *COPD* chronic obstructive pulmonary disease; *MI* myocardial infarction; *PCI* percutaneous coronary intervention. *IVC* inferior vena cava

### Surgeon Expertise and Training

Thirty studies stated how many surgeons performed RRYGB, with between one and seven surgeons participating in each study (Table 5). Whilst eight studies explicitly reported the grade of the operating surgeons, a further 22 included generic statements such as ‘experienced bariatric surgeons’. Six studies stated the number of previous RRYGB procedures performed by the participating surgeons. No studies stated any pre-specified criteria that surgeons were required to fulfil prior to participation.

**Table 5:** Summary of reporting of surgical expertise and training

	Number of studies reporting surgical expertise and training *N* = 47 *n* (%)
Number of studies reporting any information on surgical expertise and training	37 (79)
Number of studies reporting the number of surgeons involved in the study*	30 (64)
Grade of surgeon(s)	
Consultant	4 (9)
Trainee	2 (4)
Mixed consultant/trainee	2 (4)
Generic statement of experience	22 (47)
Training prior to first human procedure	
Course from device company	6 (13)
Observation by senior colleagues	1 (2)
Simulation	1 (2)
Number of prior RRYGB procedures performed by the surgeon(s)**	6 (13)
Monitoring standards of surgery during the study	
Dual-surgeon operating	3 (6)
Senior mentoring	1 (2)
Case report forms	1 (2)

* Range of number of surgeons participating per study (where reported) = 1–7

** Range of number of prior RRYGB procedures performed by surgeon(s) (where reported) = 0–153

The most common method for training surgeons was a course run by the robot manufacturer (*n* = 6). Five studies described any monitoring of surgical standards during the study: dual-surgeon operating (*n* = 3), senior mentoring (*n* = 1) and case report forms (*n* = 1).

### Technique Description

Thirty-five studies documented the model or type of surgical robot used. All studies used models Xi or Si of the Da Vinci Surgical System (Intuitive Surgical Inc., California, USA) except one study which used the Hugo™ RAS system (Medtronic, Minneapolis, MN, USA). Fifteen studies cited previous literature describing the technique of RRYGB. Fourteen unique citations were used to reference technique amongst these fifteen studies. Only three of these studies referenced the same publication, with twelve citing unique studies.

Thirty-three studies provided a written description of the technique used. Thirteen studies supplemented the written description with photographs: 10 demonstrating the robotic set-up and three demonstrating set-up and technique.

### Outcome Reporting

There were a total of 392 unique outcomes across the studies, of which 256 related to complications (Table 6). Two-hundred and thirty-four unique outcomes appeared only once across all studies. No single unique outcome was used across every study. The most common unique outcome was ‘operative time’, reported in (*n* = 41) studies. No studies described any patient-reported outcomes. One study included ‘comments from the surgeon’ but none used a formal surgeon-reported outcome measure [28]. Forty-one reported some form of economic or health resource utilisation outcome, the most common being length of stay which was reported in 38 studies.

**Table 6:** Summary of outcome reporting

Outcome Domain	Total number of outcomes	Number of unique outcomes	Number of studies reporting any outcome in this domain
Technical	174	57	45
Complications	565	256	47
Laboratory and imaging	10	4	4
Function and symptoms	79	47	19
Trends and learning curves	83	5	17
Health economic and resource utilisation	78	22	41
Patient reported	0	0	0
Surgeon reported	1	1	1
**Total**	**990**	**392**	

**Fig. 3:**
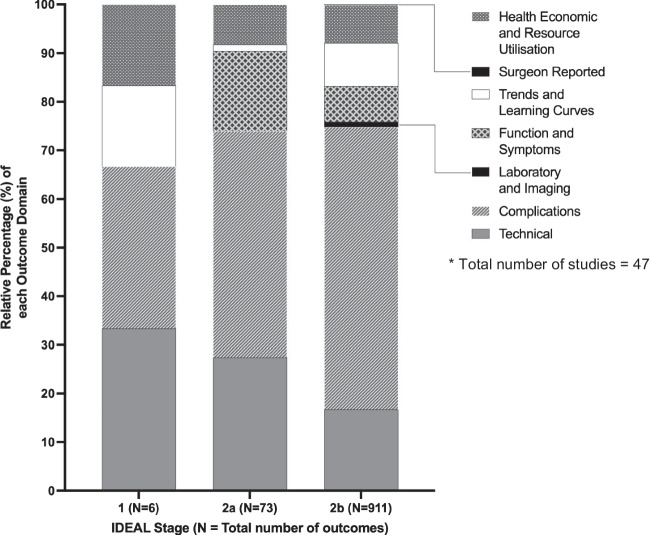
Bar chart showing reporting of outcome domains according to IDEAL stage*

Fifty-three percent of all outcomes lacked a definition and when present, definitions varied across studies. There was disparity between the outcomes that studies reported, and the recommended outcomes to be reported with respect to the study’s IDEAL stage (Fig. 3).

Seventeen studies reported trends and learning curves outcomes. There was variation in the methods used to describe the learning curve. Methods included comparing outcomes i) between intervals of cases performed (*n* = 7), ii) by case number (*n* = 7), iii) between consecutive years (*n* = 1), iv) between inexperienced and experienced surgeons (*n* = 1), and v) determining a discrete ‘number’ of cases after which surgeons were deemed to have completed their learning curve (*n* = 4).

## Discussion

This is the first systematic review to summarise and appraise how RRYGB literature has been reported. Since RRYGB was first documented in 2005, evaluation of RRYGB has not progressed sequentially through IDEAL stages, as demonstrated by the dearth of stage 2a studies, abundance of stage 2b studies and absence of RCTs. Only 35 studies reported patient selection criteria and the reporting of patient characteristics was heterogenous between studies. Few studies provided statements of ethical approval and/or conflict of interest statements. Reporting according to IDEAL guidelines was poor. Outcome selection was heterogenous and did not follow the recommendations, with 392 unique outcomes identified, 234 of which reported only once across all studies. There was a lack of information about surgeons’ experience with RRYGB and the training they received. RRYGB appears to have been adopted despite the poor reporting quality of existing studies, indicating an insufficient evidence base from which to draw meaningful conclusions about the use of RRYGB.

Three systematic reviews of observational studies comparing RRYGB and LRYGB, highlighted differences in outcomes between the two techniques [13, 69, 70]. However, all three reviews noted that the ability to make these conclusions and to compare and evaluate the safety and efficacy of literature was limited due to methodological issues across the included studies. Collectively, they noted underreporting of crucial outcome measures such as patient demographics, post-operative weight loss and follow up processes. These support our finding that the reporting quality of RRYGB literature is poor, which impedes the ability to draw meaningful conclusions about its use.

A potential solution to the problems of heterogeneous outcome selection and poor reporting of patient selection criteria and outcomes would be to create a core descriptor set and core outcome set (COS) for RRYGB as suggested by the COMET initiative [71]. This would help improve standardisation and contextualisation of study findings and facilitate evidence synthesis. Whilst a COS for all bariatric procedures has been developed, as well as a COS for the evaluation of innovative surgical procedures (COHESIVE), developing a RRYGB or robot-specific COS might improve the future evaluation of RRYGB and other robotic bariatric procedures [72, 73].

Despite the rigour with which this review was conducted, there are limitations. We excluded non-English language studies, meaning the entirety of published literature may not have been included. Four of the included studies were compared against IDEAL guidance despite being published prior to the release of the framework in 2009, which may be considered unfair. However, these studies were included to capture the breadth of available literature and better summarise and assess how RRYGB literature has been reported. As is the case with all reporting standards, the IDEAL framework has some limitations. Disparity exists between the reporting quality of RRYGB and what the IDEAL framework recommends, which may be explained by slow adoption and lack of understanding of the framework, as was demonstrated in a recent systematic review [74]. It was also challenging to implement the IDEAL algorithm for determining innovation stage. This is because most literature provided insufficient information to be able to categorise studies into either stages 2a and 2b. The IDEAL collaboration itself acknowledges uncertainty about the distinction between 2a and 2b studies [75]. There was also difficulty with classifying multicentre, large sample size, registry/database studies, due to them possessing characteristics of both stages 2a and 4. Despite its limitations, the IDEAL framework still provides the surgical community with a structured, comprehensive, and reliable approach on how to report innovative surgical research.

## Conclusion

This systematic review summarised and appraised the reporting of RRYGB as a case study of a surgical innovation. Reporting practices correlated poorly with the recommendations of the IDEAL framework across the 47 included studies. This suggests that the reporting quality of available literature is poor, impeding the ability for surgeons to draw meaningful conclusions from available evidence. This in turn complicates patients’ ability to take part in shared decision-making and give informed consent. Future studies should report findings in a structured way using the IDEAL framework or a similar reporting standard. This will ensure future research is transparent, robustly designed, prospective in nature, uses COSs and follows an ethos of incremental learning. This may reduce research waste and the premature adoption of innovative surgical procedures, ensuring their safe introduction into clinical practice.

### Supplementary Information

Below is the link to the electronic supplementary material.

Supplementary file1 (PDF 179 KB)

## Data Availability

Additional data including raw data sets are available upon request from the corresponding author.
