# Supplementary material for: A Systematic Review to Summarise and Appraise the Reporting of Surgical Innovation: a Case Study in Robotic Roux-en-Y Gastric Bypass
Source: Obes Surg. 2024 Jun 19;34(8):3058–70. doi: 10.1007/s11695-024-07329-8 (PMC11289006; doi:10.1007/s11695-024-07329-8)
Supplement: Supplementary file 1 — Supplementary file1 (PDF 179 KB) [file 11695_2024_7329_MOESM1_ESM.pdf]

Supplementary Table 1. Preferred Reporting Items for Systematic Reviews and Meta-Analyses (PRISMA) checklist[17]

Section and Topic	Item #	Checklist item	Location where item is reported
**TITLE**			
Title	1	Identify the report as a systematic review.	Title
**ABSTRACT**			
Abstract	2	See the PRISMA 2020 for Abstracts checklist.	Supplementary Table 2
**INTRODUCTION**			
Rationale	3	Describe the rationale for the review in the context of existing knowledge.	Pages 2-3
Objectives	4	Provide an explicit statement of the objective(s) or question(s) the review addresses.	Page 3
**METHODS**			
Eligibility criteria	5	Specify the inclusion and exclusion criteria for the review and how studies were grouped for the syntheses.	Pages 3-5
Information sources	6	Specify all databases, registers, websites, organisations, reference lists and other sources searched or consulted to identify studies. Specify the date when each source was last searched or consulted.	Page 3
Search strategy	7	Present the full search strategies for all databases, registers and websites, including any filters and limits used.	Supplementary Table 3
Selection process	8	Specify the methods used to decide whether a study met the inclusion criteria of the review, including how many reviewers screened each record and each report retrieved, whether they worked independently, and if applicable, details of automation tools used in the process.	Pages 3-4
Data collection process	9	Specify the methods used to collect data from reports, including how many reviewers collected data from each report, whether they worked independently, any processes for obtaining or confirming data from study investigators, and if applicable, details of automation tools used in the process.	Page 4
Data items	10a	List and define all outcomes for which data were sought. Specify whether all results that were compatible with each outcome domain in each study were sought (e.g. for all measures, time points, analyses), and if not, the methods used to decide which results to collect.	Pages 4-5<br>Table 1

Section and Topic	Item #	Checklist item	Location where item is reported
	10b	List and define all other variables for which data were sought (e.g. participant and intervention characteristics, funding sources). Describe any assumptions made about any missing or unclear information.	Pages 4-5
Study risk of bias assessment	11	Specify the methods used to assess risk of bias in the included studies, including details of the tool(s) used, how many reviewers assessed each study and whether they worked independently, and if applicable, details of automation tools used in the process.	NA*
Effect measures	12	Specify for each outcome the effect measure(s) (e.g. risk ratio, mean difference) used in the synthesis or presentation of results.	NA*
Synthesis methods	13a	Describe the processes used to decide which studies were eligible for each synthesis (e.g. tabulating the study intervention characteristics and comparing against the planned groups for each synthesis (item #5)).	NA*
	13b	Describe any methods required to prepare the data for presentation or synthesis, such as handling of missing summary statistics, or data conversions.	NA*
	13c	Describe any methods used to tabulate or visually display results of individual studies and syntheses.	NA*
	13d	Describe any methods used to synthesize results and provide a rationale for the choice(s). If meta-analysis was performed, describe the model(s), method(s) to identify the presence and extent of statistical heterogeneity, and software package(s) used.	Page 5
	13e	Describe any methods used to explore possible causes of heterogeneity among study results (e.g. subgroup analysis, meta-regression).	NA*
	13f	Describe any sensitivity analyses conducted to assess robustness of the synthesized results.	NA*
Reporting bias assessment	14	Describe any methods used to assess risk of bias due to missing results in a synthesis (arising from reporting biases).	NA*
Certainty assessment	15	Describe any methods used to assess certainty (or confidence) in the body of evidence for an outcome.	NA*
**RESULTS**			
Study selection	16a	Describe the results of the search and selection process, from the number of records identified in the search to the number of studies included in the review, ideally using a flow diagram.	Page 5<br>Figure 1
	16b	Cite studies that might appear to meet the inclusion criteria, but which were excluded, and explain why they were excluded.	Figure 1

Section and Topic	Item #	Checklist item	Location where item is reported
Study characteristics	17	Cite each included study and present its characteristics.	Pages 5-6<br>Table 2<br>Supplementary Table 4
Risk of bias in studies	18	Present assessments of risk of bias for each included study.	NA*
Results of individual studies	19	For all outcomes, present, for each study: (a) summary statistics for each group (where appropriate) and (b) an effect estimate and its precision (e.g. confidence/credible interval), ideally using structured tables or plots.	NA*
Results of syntheses	20a	For each synthesis, briefly summarise the characteristics and risk of bias among contributing studies.	Pages 5-8<br>Table 3<br>Table 4<br>Table 5<br>Table 6<br>Figure 2<br>Figure 3
	20b	Present results of all statistical syntheses conducted. If meta-analysis was done, present for each the summary estimate and its precision (e.g. confidence/credible interval) and measures of statistical heterogeneity. If comparing groups, describe the direction of the effect.	NA*
	20c	Present results of all investigations of possible causes of heterogeneity among study results.	NA*
	20d	Present results of all sensitivity analyses conducted to assess the robustness of the synthesized results.	NA*
Reporting biases	21	Present assessments of risk of bias due to missing results (arising from reporting biases) for each synthesis assessed.	NA*
Certainty of evidence	22	Present assessments of certainty (or confidence) in the body of evidence for each outcome assessed.	NA*
**DISCUSSION**			
Discussion	23a	Provide a general interpretation of the results in the context of other evidence.	Pages 8-9

Section and Topic	Item #	Checklist item	Location where item is reported
	23b	Discuss any limitations of the evidence included in the review.	Page 9
	23c	Discuss any limitations of the review processes used.	Page 9
	23d	Discuss implications of the results for practice, policy, and future research.	Pages 8-9
**OTHER INFORMATION**			
Registration and protocol	24a	Provide registration information for the review, including register name and registration number, or state that the review was not registered.	Title page
	24b	Indicate where the review protocol can be accessed, or state that a protocol was not prepared.	Page 3-4
	24c	Describe and explain any amendments to information provided at registration or in the protocol.	NA*
Support	25	Describe sources of financial or non-financial support for the review, and the role of the funders or sponsors in the review.	Title page
Competing interests	26	Declare any competing interests of review authors.	Title page
Availability of data, code and other materials	27	Report which of the following are publicly available and where they can be found: template data collection forms; data extracted from included studies; data used for all analyses; analytic code; any other materials used in the review.	On request

*****The current review is presented as a narrative synthesis of all studies reporting outcomes of robotic Roux-en-Y gastric bypass. Some points are therefore, not applicable.

Supplementary Table 2. Preferred Reporting Items for Systematic Reviews and Meta-Analyses (PRISMA) for abstracts checklist[17].

Section and Topic	Item #	Checklist item	Reported (Yes/No)
**TITLE**			
Title	1	Identify the report as a systematic review.	Yes
**BACKGROUND**			
Objectives	2	Provide an explicit statement of the main objective(s) or question(s) the review addresses.	Yes
**METHODS**			
Eligibility criteria	3	Specify the inclusion and exclusion criteria for the review.	Yes
Information sources	4	Specify the information sources (e.g. databases, registers) used to identify studies and the date when each was last searched.	Yes
Risk of bias	5	Specify the methods used to assess risk of bias in the included studies.	No*
Synthesis of results	6	Specify the methods used to present and synthesise results.	Yes
**RESULTS**			
Included studies	7	Give the total number of included studies and participants and summarise relevant characteristics of studies.	Yes
Synthesis of results	8	Present results for main outcomes, preferably indicating the number of included studies and participants for each. If meta-analysis was done, report the summary estimate and confidence/credible interval. If comparing groups, indicate the direction of the effect (i.e. which group is favoured).	Yes
**DISCUSSION**			
Limitations of evidence	9	Provide a brief summary of the limitations of the evidence included in the review (e.g. study risk of bias, inconsistency and imprecision).	Yes
Interpretation	10	Provide a general interpretation of the results and important implications.	Yes
**OTHER**			
Funding	11	Specify the primary source of funding for the review.	Title page
Registration	12	Provide the register name and registration number.	Title page

*Not applicable as this review assesses the quality of reporting rather than the risk of bias

Supplementary Table 3. Search strategy

Ovid (Medline/Embase)

1 ((Robot* adj (RYGB or ((bariatric or stomach) adj surg*))) or RRYGB).mp. (177)
2 robotics/ (58877)
3 robot assisted surgery/ (10072)
4 robotic surgical procedure/ or robotic surgical procedures/ (10745)
5 computer assisted surgery/ or surgery, computer-assisted/ (26544)
6 computer assisted surgery system/ (123)
7 robotic surgical device/ or robotic console/ or robotic navigation system/ or robotic surgical system/ or robotic catheter system/ or robotic neurological surgical equipment/ or robotic orthopedic surgical system/ or robotic hip surgery system/ or robotic knee surgery system/ or robotic spine surgical system/ (2146)
8 robotic surgical instrument/ or robotic endoscope/ or robotic needle driver/ or robotic retractor/ or robotic scissors/ or robotic forceps/ or robotic dissecting forceps/ or robotic grasping forceps/ or robotic sealing device/ or robotic stapler/ or robotic vessel sealer/ (238)
9 robot assisted.hw. (12893)
10 (robot* or telerobot* or tele-robot* or telesurger* or tele-surger*).ti,ab,kw. (113136)
11 (computer assist* adj5 (operation* or procedur* or surger* or surgeon? or surgical*)).ti,ab,kw. (5832)
12 (da Vinci? or davinci?).mp. (11864)
13 (remote adj2 (operation* or procedur* or surger* or surgeon? or surgical*)).ti,ab,kw. (1500)
14 ("state of the art" adj (surger* or surgical*)).ti,ab,kw. (216)
15 (Arthrobot* or Aesop* or Hugo* or Mako* or MiroSurge* or Monarch* or Neuroarm* or Probot* or Revo-I or ROBODOC).mp. (7915)
16 (Senhance* or Sina or Sofie or SPIDER or SPORT or SurgiBot or Telelap or Unimation or Puma or Versius or ZEUS).mp. (169589)
17 or/2-16 (329928)
18 Anastomosis, Roux-en-Y/ (12158)
19 Roux-en-y Gastric Bypass/ (15963)
20 roux-en-y.mp. (33458)
21 rouxeny.mp. (15)
22 RYGB.ti,ab,kf,kw. (9667)
23 *gastric bypass surgery/ (1540)
24 ((stomach or gastric) adj bypass).ti,ab,kf,kw. (34002)
25 stomach stapl*.ti,ab,kf,kw. (46)
26 duodenal switch*.ti,ab,kf,kw. (2394)
27 (anastomo* adj3 surg* adj3 (stomach or gastric)).ti,ab,kf,kw. (144)
28 MBSAQIP.mp. (493)
29 or/18-28 (52523)
30 17 and 29 (1143)
31 1 or 30 (1175)
32 (conference abstract or conference review).pt. (3786108)
33 31 not 32 (689)
34 remove duplicates from 33 (420)

Cochrane Library

#1 ((robot* or telerobot* or tele-robot* or telesurger* or tele-surger* or (computer assisted near/3 surgery) or (computer assist* NEAR (operation* or procedur* or surger* or surgeon? or surgical*)) or (remote NEAR/2 (operation* or procedur* or surger* or surgeon? or surgical*)) or ("state of the art" NEXT (surger* or surgical*)) or “da Vinci*” or davinci* or Arthrobot* or Aesop* or Hugo* or Mako* or MiroSurge* or Monarch* or Neuroarm* or Probot* or Revo-I or ROBODOC or Senhance* or Sina or Sofie or SPIDER or SPORT or SurgiBot or Telelap or Unimation or Puma or Versius or ZEUS) AND (roux-en-y or rouxeny or RYGB or RRYGB or ((stomach or gastric) NEAR/3 bypass) or (stomach next stapl*) or (duodenal next switch*) or (anastomo* NEAR surg* NEAR (stomach or gastric)) or MBSAQIP)):ti,ab,kw
#2 (robot* near/3 ((bariatric or stomach) near/3 surg*)):ti,ab,kw
#3 (#1 OR #2)

Web of Science

(TS=(robot* or telerobot* or tele-robot* or telesurger* or tele-surger* or ("computer assist*" NEAR (operation* or procedur* or surger* or surgeon* or surgical*)) or (remote NEAR (operation* or procedur* or surger* or surgeon* or surgical*)) or ("state of the art" NEXT (surger* or surgical*)) or "da Vinci*" or davinci* or Arthrobot* or Aesop* or Hugo* or Mako* or MiroSurge* or Monarch* or Neuroarm* or Probot* or Revo-I or ROBODOC or Senhance* or Sina or Sofie or SPIDER or SPORT or SurgiBot or Telelap or Unimation or Puma or Versius or ZEUS)) AND (TS=(roux-en-y or rouxeny or RYGB or RRYGB or ((stomach or gastric) NEAR bypass)) OR TS=("stomach stapl*" or "duodenal switch*" or MBSAQIP) OR TS=(anastomo* NEAR surg* NEAR (stomach or gastric)) OR TS=(robot* NEAR (bariatric or stomach) NEAR surg*))

Supplementary Table 4. General characteristics of included studies

First author	Year	Country	Temporality of data collection	Study design	IDEAL stage	Number of participants	Number of comparison arms	Comparison arms (number of participants per arm)	Single or multi Centre
Celio[23]	2017	United States	Mixture of retrospective and prospective	Comparative	2b	137,455	1	RRYGB (2415)<br>LRYGB (135,040)	Multi Centre
Lyn-Sue[25]	2016	United States	Retrospective	Comparative	2b	50	1	RRYGB (25)<br>LRYGB (25)	Single Centre
Aggarwal[26]	2015	India	Not reported	Case Report	2a	1	0	RRYGB (1)	Single Centre
Ahmad[15]	2016	United States	Retrospective	Comparative	2b	345	1	RRYGB (172)<br>LRYGB (173)	Single Centre
Ayloo[27]	2016	United States	Retrospective	Comparative	2b	192	2	RRYGB (61)<br>LRYGB (46)<br>Hybrid RRYGB (85)	Single Centre
Mohr[7]	2005	United States	Mixture of retrospective and prospective	Comparative	1	20	1	RRYGB (10)<br>LRYGB (10)	Single Centre
Renaud[28]	2013	France	Prospective	Case Series	2b	154	0	RRYGB (154)	Not Reported
Rogula[29]	2018	United States	Retrospective	Comparative	2b	578	3	RRYGB (103)<br>Circular Stapled GJA LRYGB (125)<br>Linear Stapled GJA LRYGB (300)<br>Hand-sewn GJA LRYGB (50)	Single Centre

First author	Year	Country	Temporality of data collection	Study design	IDEAL stage	Number of participants	Number of comparison arms	Comparison arms (number of participants per arm)	Single or multi Centre
Lundberg [12]	2018	United States	Retrospective	Comparative	2b	39,425	1	RRYGB (2822)<br>LRYGB (36,603)	Multi Centre
Diamantis [30]	2010	Greece	Not reported	Case Series	2a	9	0	RRYGB (9)	Not Reported
Hagen [31]	2012	Switzerland	Mixture of retrospective and prospective	Comparative	2b	990	2	RRYGB (143)<br>Open RYGB (524)<br>LRYGB (323)	Single Centre
Scozzari [32]	2014	Italy	Not reported	Comparative	2b	150	1	RRYGB (48)<br>LRYGB (102)	Single Centre
Ayloo [33]	2014	United States	Retrospective	Case Series	2b	61	0	RRYGB (61)	Single Centre
Buchs [34]	2014	Switzerland	Prospective	Comparative	2b	777	1	RRYGB (388)<br>LRYGB (389)	Single Centre
Hubens [35]	2007	Netherlands	Not reported	Comparative	2b	90	1	RRYGB (45)<br>LRYGB (45)	Single Centre
Beckmann [36]	2020	Germany	Retrospective	Comparative	2b	222	1	RRYGB (114)<br>LRYGB (108)	Single Centre
Park [37]	2011	United States	Retrospective	Comparative	2b	300	1	RRYGB (105)<br>LRYGB (195)	Single Centre
Kim [10]	2011	United States	Not reported	Case Series	2b	271	0	RRYGB (271)	Not Reported
Benizri [38]	2013	France	Prospective	Comparative	2b	200	1	RRYGB (100)<br>LRYGB (100)	Single Centre

First author	Year	Country	Temporality of data collection	Study design	IDEAL stage	Number of participants	Number of comparison arms	Comparison arms (number of participants per arm)	Single or multi Centre
Mohr [39]	2006	United States	Retrospective	Case Series	2a	75	0	RRYGB (75)	Single Centre
Bustos [40]	2019	United States	Retrospective	Case Series	2b	67	0	RRYGB (67)	Single Centre
Sharma [41]	2018	United States	Retrospective	Comparative	2b	23,940	1	RRYGB (2660)<br>LRYGB (21,280)	Not Reported
Smeenk [42]	2016	Netherlands	Retrospective	Comparative	2b	200	1	RRYGB (100)<br>LRYGB (100)	Single Centre
Curet [43]	2009	United States	Retrospective	Comparative	2b	135	2	RRYGB (21)<br>Stapled GJA LRYGB (78)<br>Hand-sewn GJA LRYGB (36)	Single Centre
Starnes [44]	2015	United States	Retrospective	Case Series	2b	163	0	RRYGB (163)	Single Centre
Hagen [45]	2017	Switzerland	Retrospective	Comparative	2b	98	1	RRYGB (49)<br>RRYGB EWSS System (49)	Single Centre
Tieu [46]	2013	United States	Retrospective	Case Series	2b	1100	0	RRYGB (1100)	Multi Centre
Stefanidis [47]	2017	United States	Retrospective	Comparative	2b	246	1	RRYGB (125)<br>LRYGB (121)	Single Centre
Wood [46]	2013	United States	Retrospective	Comparative	2b	200	1	RRYGB (100)<br>LRYGB (100)	Single Centre
Bindal [48]	2015	United States	Retrospective	Comparative	2b	200	1	RRYGB (82)<br>Hybrid RYGB (118)	Single Centre

Author	Year	Country	Data Collection	Study Design	IDEAL Stage	Number of Participants	Number of Comparison arms	Comparison Arms (participants per arm)	Single or Multi Centre
Buchs [49]	2016	Switzerland	Retrospective	Comparative	2b	214	2	RRYGB (65)<br>LRYGB (54)<br>Open RYGB (95)	Single Centre
Roriz-Silva [54]	2022	Spain	Retrospective	Comparative	2b	466	2	RRYGB (233)<br>LRYGB (233)	Single Centre
Dudash [55]	2022	United States	Retrospective	Comparative	2b	573	2	RRYGB (111)<br>LRYGB (462)	Single Centre
Bedirli [56]	2022	Turkey	Retrospective	Case Series	2a	527	1	RRYGB (527)	Not Reported
Morrell [57]	2021	Brazil	Retrospective	Case Series	2a	329	1	RRYGB (329)	Not Reported
Beckmann [58]	2020	Germany	Retrospective	Comparative	2b	222	2	RRYGB (114)<br>LRYGB (108)	Single Centre
King [59]	2020	United States	Retrospective	Comparative	2b	162	2	RRYGB (26)<br>LRYGB(136)	Single Centre
Lainas [60]	2021	France	Prospective	Comparative	2b	161	2	RRYGB (100)<br>LRYGB (61)	Single Centre
Oviedo [61]	2021	United States	Retrospective	Comparative	2b	100	2	RRYGB (50)<br>LRYGB (50)	Single Centre
Iranmesh [62]	2023	United States/ Switzerland	Retrospective	Comparative	2b	1276	1	RRYGB (1276)	Multi Centre
Barros [63]	2023	Brazil	Retrospective	Comparative	2b	221	2	RRYGB (121)<br>LRYGB (100)	Single Centre

Author	Year	Country	Data Collection	Study Design	IDEAL Stage	Number of Participants	Number of Comparison arms	Comparison Arms (participants per arm)	Single or Multi Centre
Kauffels [64]	2023	Germany	Retrospective	Comparative	2b	113	2	RRYGB BMI >50 (72)<br>RRYGB BMI <50 (41)	Single Centre
Rafaelli [65]	2024	Italy	Retrospective	Case Series	2a	15	1	RRYGB (15)	Single Centre
Rahimi [66]	2023	United States	Retrospective	Comparative	2b	190,553*	2	RRYGB (19087)<br>LRYGB (171,466)	Multi Centre
Sebastian [67]	2021	United States	Retrospective	Comparative	2b	13,576*	2	RRYGB (1502)<br>LRYGB (12,074)	Multi Centre
Edwards [68]	2022	United States	Retrospective	Comparative	2b	157,716*	3	RRYGB Black non-Hispanic (23,938)<br>RRYGB White Non-Hispanic (111,391)<br>Hispanic (22,387)	Multi Centre
Sebastian [69]	2018	United States	Retrospective	Comparative	2b	79,429*	2	RRYGB (5,780<br>LRYGB (73,649)	Multi Centre

Key: RRYGB, Robotic Roux-en-Y Gastric Bypass; LRYGB, Laparoscopic Roux-en-Y Gastric Bypass; Hybrid RYGB, Roux-en-Y Gastric Bypass using a combination of both laparoscopic and robotic approaches; GJA, Gastrojejunal Anastomosis; EWSS, Endo Wrist Stapling System

*Total number of participants who underwent RRYGB or LRYGB, participants who underwent index procedures other than RRYGB/LRYGB were excluded from the number
